# A Study on the Biological Activity of Optically Pure Aziridine Phosphines and Phosphine Oxides

**DOI:** 10.3390/molecules29071430

**Published:** 2024-03-22

**Authors:** Aleksandra Kowalczyk, Adam M. Pieczonka, Hassan Kassassir, Michał Rachwalski, Paweł Stączek

**Affiliations:** 1 Department of Molecular Microbiology, Faculty of Biology and Environmental Protection, University of Lodz, Banacha 12/16, 90-237 Lodz, Poland; pawel.staczek@biol.uni.lodz.pl; 2 Department of Organic and Applied Chemistry, Faculty of Chemistry, University of Lodz, Tamka 12, 91-403 Lodz, Poland; michal.rachwalski@chemia.uni.lodz.pl; 3 Cellular Signalling Laboratory, Institute for Medical Biology, Polish Academy of Sciences, Lodowa 106, 93-232 Lodz, Poland; hkassassir@cbm.pan.pl

**Keywords:** aziridines, biological activity, cytotoxicity, organophosphorus compounds

## Abstract

A series of optically pure aziridine phosphines and their corresponding phosphine oxides were synthesized through established chemical methodologies. The compounds were systematically investigated for their biological properties. Notably, all synthesized compounds demonstrated moderate antibacterial activity only against the reference strain of *Staphylococcus aureus*. However, compounds **5** and **7** exhibited noteworthy cell viability inhibition of human cervical epithelioid carcinoma HeLa cells and endometrial adenocarcinoma Ishikawa cells. Further studies of these compounds revealed additional biological effects, including disruption of the cell membrane in high concentrations, cell cycle arrest in the S phase, and the induction of reactive oxygen species (ROS). Comparative analysis of the two classes of chiral organophosphorus derivatives of aziridines indicated that chiral phosphine oxides displayed significantly higher biological activity. Consequently, these findings suggest that chiral phosphine oxides may be potential candidates for the development of anticancer drugs. In light of the significant interest in preparations whose structure is based on a three-membered aziridine ring in terms of potential anticancer therapy, this research fits into the current research trend and should constitute a valuable addition to the current state of knowledge and the existing library of aziridine derivatives with anticancer properties.

## 1. Introduction

Aziridines constitute three-membered, strained, saturated, heterocyclic compounds [1]. These reactive compounds have an extremely wide spectrum of applications in many fields of science [2]. Over forty years ago, our research group discovered that aziridines perfectly complex with ions of zinc and other metals [3,4]. The results of these early studies turned out to be the beginning of our research work on the effective application of chiral aziridine catalysts in asymmetric synthesis involving various metals [5]. For example, the reactions of aldol condensation and the addition of diethylzinc to aldehydes and enones took place in the presence of aziridine catalysts with high chemical yields and a very high degree of enantioselectivity [5]. 

**Figure 1:**
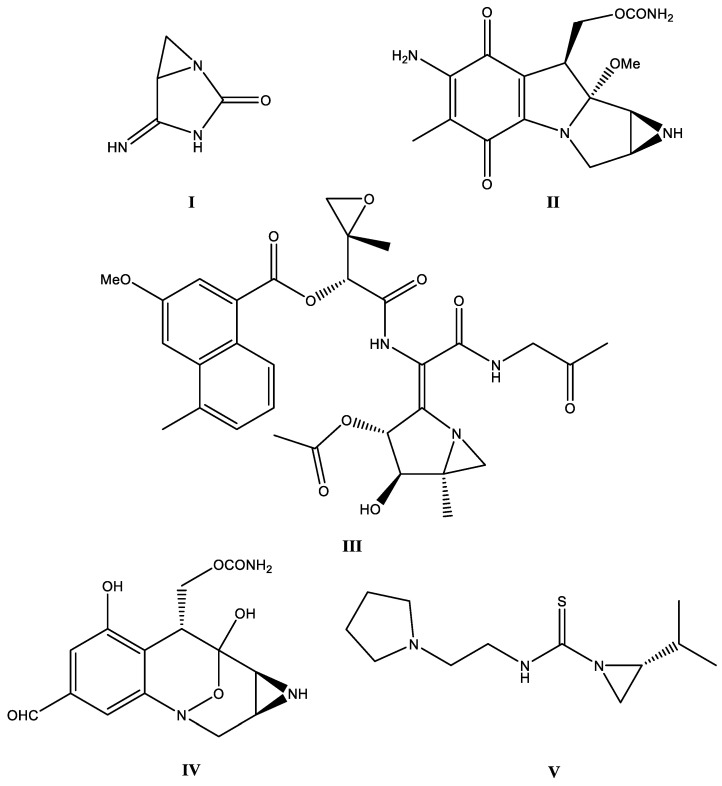
Examples of aziridine derivatives exhibiting biological activity.

On the other hand, aziridine derivatives also exhibit a number of interesting biological activities [6,7]. Among numerous examples of compounds of this type, one can distinguish imexon (**I**) [8], which belongs to a family of drugs called cyanoaziridine derivatives. It is a small molecule that induces mitochondrial oxidation and a loss of membrane potential and cytochrome C, leading to apoptosis [9]. Other examples are mitomycin C (**II**), azinomycin A (**III**) [10,11,12], and FR900482 (**IV**) [13] (Figure 1). Mitomycin C and azinomycin A are known as antitumor drugs and both processes lead to the inhibition of DNA replication. FR900482 is a close cousin of mitomycin C that exploits DNA cross-linking, while imexon is an antitumor agent that has selective activity in multiple myeloma but is also effective in the case of other cancer cell lines. The two latest examples are not yet registered drugs; they are still in the clinical trial stage. In 2018, our research group reported the synthesis and in vitro studies of the antimicrobial activity of aziridine-thiourea derivatives (**V**) [14]. Some of those compounds exhibited promising activity towards *Escherichia coli*, *Staphylococcus epidermidis*, and clinical isolates of *Staphylococcus aureus*. The most bactericidal aziridine derivatives also showed the highest toxic activity against both tested cell lines with no selectivity for tumor cells. Recently, we also reported aziridine–hydrazide hydrazone derivatives with antineoplastic potential against glioblastoma cells [15]. Although, in the chemical literature, there are numerous examples of research on the biological activity (including anticancer) of phosphines and phosphine oxides, there are slightly fewer reports on biological research on systems based on the aziridine skeleton; however, systems with both mentioned structural motifs in their structure are practically non-existent in the literature (only Dogan’s work on biological research on aziridine phosphonates deserves mention [7]).

Continuing our research on the therapeutic potential of aziridine derivatives, we decided to verify the antibacterial and anticancer activity of the previously synthesized aziridine phosphines [16] and aziridine phosphine oxides [17] in L929, HeLa, and Ishikawa cell lines. Based on our previous studies, the inclusion of an additional polar subunit increases the biological activity of aziridine derivatives [14,15]. Organophosphorus compounds have been intensively investigated but, surprisingly, only few reports on the in vitro antiproliferative properties of phosphine oxides and phosphines are available today [18,19,20]. Phosphine oxides are highly polar functional groups leading to very high aqueous solubility and metabolic stability, which should increase the anticancer activity of aziridine derivatives. 

## 2. Results and Discussion

### 2.1. Chemistry

**Figure 2:**
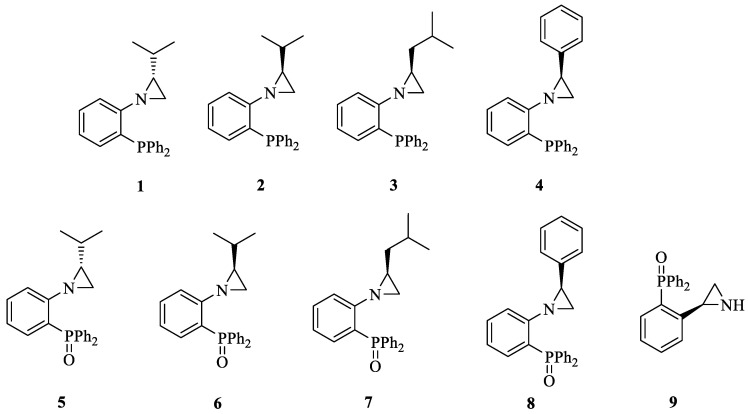
The structure of the tested aziridine derivatives.

At the beginning, a series of aziridine phosphines (**1**–**4**) and aziridine phosphine oxides (**5**–**8**) bearing a series of chiral substituents in the aziridine ring and a triphenylphosphine moiety was synthesized (Figure 2). The choice of the triphenylphosphine group was dictated by data from the literature indicating the high biological activity of aromatic phosphines and phosphine oxides [18,19] as well as good availability of the starting materials. The choice of substituents in the aziridine ring resulted from our previous studies indicating the high activity of derivatives with a branched aliphatic substituent in a precisely defined absolute configuration (exclusively (*S*)-isopropyl group) [14]. Additionally, we decided to check the activity of an analogous compound with an unprotected amino group in NH-aziridine (**9**). 

### 2.2. Biology

#### 2.2.1. Antibacterial Activity

A panel of reference bacteria, including *Escherichia coli* NCTC 8196, *Staphylococcus aureus* ATCC 6538, *Klebsiella pneumoniae* ATCC 13883, and *Pseudomonas aeruginosa* NCTC 6749, was used to assess the antibacterial activity of the nine tested aziridine phosphines (**1**–**4**) and aziridine phosphine oxides (**5**–**9**). The two-fold microdilution method, following EUCAST guidelines, was used to determine a minimal inhibitory concentration (MIC) of each compound, as presented in Table S1. Within the tested concentration range of 3.125–100 µM, all aziridine derivatives exhibited activity only against *S. aureus*, with an MIC value of 50 µM. The remaining bacterial strains were insensitive to all tested compounds, with MIC values exceeding 100 µM. Notably, it is worth mentioning that these strains are Gram-negative bacteria, which suggests that the limited activity observed may be attributed to the presence of an outer membrane, hindering the penetration of aziridine derivatives. Considering the moderate activity against the reference strain of *S. aureus*, the antibacterial effect of the tested compounds was further assessed against a panel of 12 clinical *S. aureus* strains obtained from various sources, including the naso-pharynx (C4, C7, C8, C19), ulcers and furuncles (D12, F1, F7, F12), and bones (D14, D15, D17, D120). Only in the case of three aziridine phosphine oxides (**5**, **6**, **7**) was it possible to determine the MIC value at the highest tested concentration equal to 100 µM. Importantly, all clinical isolates of *S. aureus* demonstrated increased resistance to the tested aziridine derivatives compared to the reference strain (Table S2).

#### 2.2.2. Cell Viability Inhibition

Considering our previous research and the literature reports on the antitumor activity of aziridine derivatives, aziridine phosphines (**1**–**4**) and aziridine phosphine oxides (**5**–**9**) were tested to investigate their potential anticancer activity. The in vitro effects on cell viability were assessed using the 3-(4,5-dimethylthiazol-2-yl)-2,5-diphenyltetrazolium bromide (MTT) assay on non-tumorigenic murine fibroblasts (L929) as well as two cancer cell lines: human cervical epithelioid carcinoma (HeLa) cells and endometrial adenocarcinoma (Ishikawa) cells. The half-maximal inhibitory concentration (IC_50_) values were determined for each aziridine derivative within the tested concentration range of 3.125–100 µM, with at least one cell line exhibiting measurable activity (Table 1). Generally, the aziridine phosphine oxides displayed higher activity compared to the aziridine phosphines. The highest cell viability inhibition was observed in the case of two aziridine phosphine oxides, **5** and **7**. They significantly decreased the cell viability of both cancer cell lines, HeLa and Ishikawa, with IC_50_ values of 6.4 µM and 4.6 µM, respectively, for compound **5** and 7.1 µM and 10.5 µM, respectively, for compound **7**. Importantly, the IC_50_ values obtained for compounds **5** and **7** on both cancer cell lines were comparable to the well-known anticancer drug cisplatin. Only in the case of normal L929 and cancerous Ishikawa cells, the IC_50_ values for compound **5** were significantly lower than for cisplatin. These two compounds also exhibited a selectivity index (SI) exceeding one for both HeLa and Ishikawa cells, indicating a greater efficacy against cancerous cells compared to normal fibroblasts. Nevertheless, the SI values were moderately elevated, reaching 1.7 and 2.3 for **5** and 2.5 and 1.7 for **7**. However, the SI values for the widely used anticancer drug cisplatin closely matched those of compounds **5** and **7**. Furthermore, compounds **1** and **2** demonstrated the highest selectivity, attaining SI values of 4.9 and 6.7, respectively, albeit exclusively against Ishikawa cells.

#### 2.2.3. LDH Release Assay

**Figure 3:**
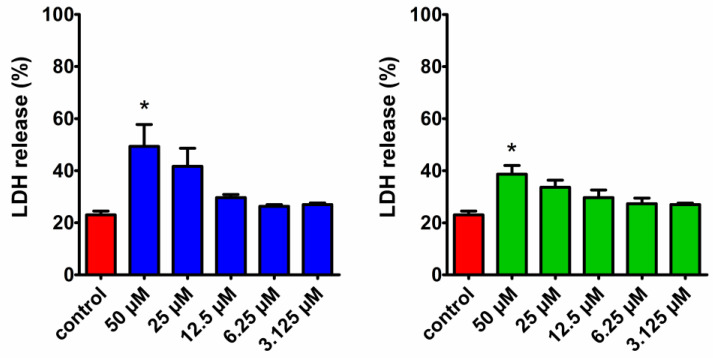
The effects of **5** (blue) and **7** (green) on LDH release from HeLa cells. The cells were treated with compounds at a concentration range of 3.125–50 μM for 24 h at 37 °C and 5% CO_2_. Data are presented as the mean LDH release (%) ± SD of two experiments compared to untreated control (red). Statistical significance measured by two-way analysis of variance (ANOVA) is indicated by asterisks: (*) *p* < 0.05.

Among all the tested aziridine phosphines and aziridine phosphine oxides, compounds **5** and **7** exhibited the most promising cell viability inhibition towards cancer cells and were used for further investigation. In order to distinguish whether the cell viability inhibition was attributed to growth inhibition or cytotoxicity manifested by cell membrane disruption, the lactate dehydrogenase (LDH) release assay was conducted. LDH is a cytoplasmic enzyme rapidly released from cells upon membrane damage, a key feature of apoptosis, necrosis, and other forms of cell death. The released enzyme can be detected due to its ability to convert lactate into pyruvate and NADH, which is then specifically determined by a colorimetric assay. The assay revealed that LDH release from HeLa cells after a 24 h treatment with **5** and **7** was concentration-dependent (Figure 3). Both compounds induced a significant LDH release compared to the untreated control, but only at the highest concentration of 50 µM. Notably, at concentrations close to the IC_50_ values determined in the MTT assay (6.4 µM for **5** and 7.1 µM for **7**), the level of LDH released was similar to the control, suggesting that the observed decrease in cell viability was due to the inhibition of cell growth rather than membrane disruption.

#### 2.2.4. Cell Cycle Analysis

**Figure 4:**
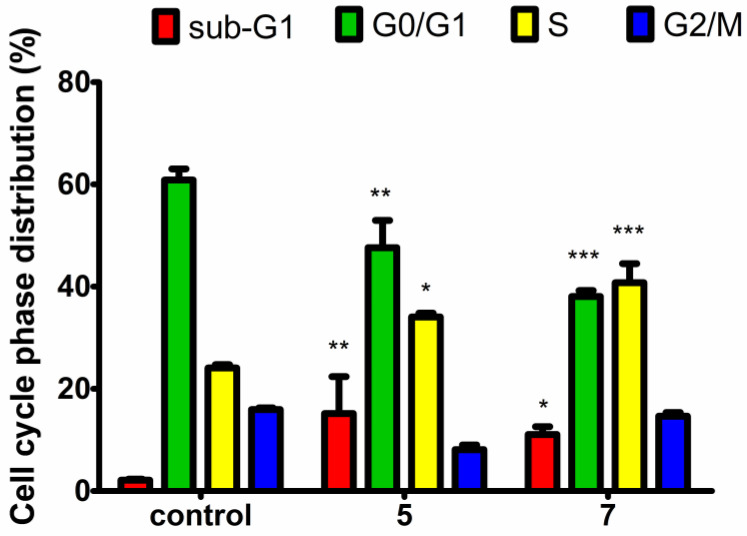
Effects of **5** and **7** on cell cycle phase distribution in HeLa cells compared to untreated control. The cells were treated for 24 h at 37 °C and 5% CO_2_ with compounds at a concentration corresponding to their IC_50_ values. Data are expressed as mean ± SD of two independent experiments. Statistical significance in comparison with the untreated control, measured by two-way analysis of variance (ANOVA) followed by Bonferroni multiple comparison test, is indicated by asterisks: (*) *p* < 0.05, (**) *p* < 0.01, (***) *p* < 0.001.

The MTT and LDH release assays provided evidence that aziridine phosphine oxides **5** and **7** inhibit cancer cell viability at concentrations equal to their respective IC_50_ values through antiproliferative mechanisms rather than by inducing cell death. It is expected that such observations will be reflected in cell cycle disturbances. To investigate which phase of the cell cycle is arrested, a cell cycle analysis was performed using propidium iodide (PI) staining. PI binds in proportion to the amount of DNA present in the cell. As a result of DNA replication, the cells in the S phase of the cell cycle contain more DNA than cells in the G1 phase. Furthermore, cells in the G2 phase, after DNA replication and prior to mitosis, exhibit approximately twice the DNA content and thus appear brighter compared to G1-phase cells. Before the exact analysis of the obtained results, we utilized a pre-gate to define the counted cells to be analyzed for cell cycle distribution. In the gating procedure, we excluded the population of objects with the smallest sizes (representing cellular debris) and cell clusters (which can exhibit higher levels of fluorescence) and we included only the main populations of cells. Based on the analysis of DNA content, it was found that both tested aziridine phosphine oxides caused a significant cell arrest in the S phase during replication, concomitantly reducing the number of cells in the G1 phase (Figure 4). These findings confirmed the antiproliferative activity of these compounds. The disruption of the cell cycle and the level of S-phase arrest were comparable between **5** and **7**, indicating their similar activity and closely matched IC_50_ values. However, the histograms of cell cycle distribution indicated that compound **5** caused cell arrest mainly in the early stages of the S phase, while compound **7** was more effective in the late S phase. Moreover, both compounds significantly increased the number of cells in the sub-G1 phase, indicative of apoptosis, suggesting an additional mechanism of the tested aziridine phosphine oxides contributing to the decreased viability of HeLa cells, alongside their antiproliferative effect. 

#### 2.2.5. Reactive Oxygen Species Measurement

To investigate whether additional mechanisms may underlie the inhibitory effect of compounds **5** and **7** on cancer cell viability, their ability to induce the generation of reactive oxygen species (ROS) in HeLa cells was assessed. Reactive oxygen species have the potential to induce deleterious effects on cellular components, leading to damage in DNA, proteins, and cell membranes, and ultimately resulting in a reduction in cell viability.

**Figure 5:**
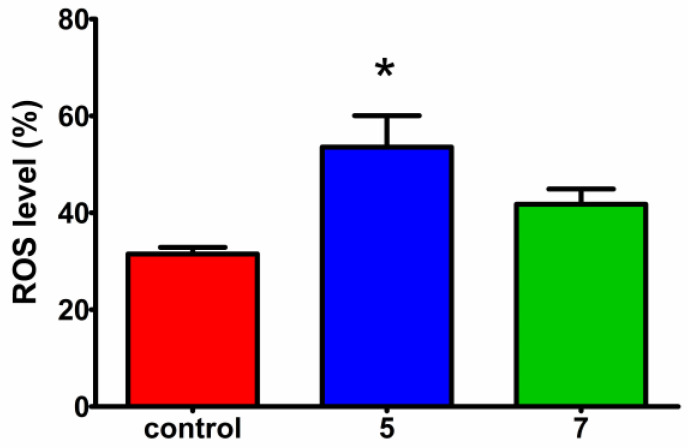
Intracellular ROS generation in HeLa cells after treatment with **5** (blue) and **7** (green) for 24 h at 37 °C and 5% CO_2_. The concentration for each compound corresponded to the IC_50_ values. Oxidative stress was evaluated using H2DCFDA by flow cytometry. Data are expressed as the mean ± SD of two experiments. Statistical significance in comparison with the untreated control, measured by two-way analysis of variance (ANOVA) followed by Bonferroni multiple comparison test, is indicated by asterisks: (*) *p* < 0.05.

The intracellular ROS levels were evaluated using 2′,7′-dichlorofluorescein diacetate (H2DCFDA) staining. This cell membrane-permeable probe is deacetylated by cellular esterases to a non-fluorescent compound, which is then oxidized by ROS to 2′,7′-dichlorofluorescein (DCF), which remains in cells, is not removed from them, and emits strong fluorescence that can be quantified using a flow cytometer. To evaluate oxidative stress, HeLa cells were treated with the respective compound at a concentration equal to its IC_50_ value for 24 h, followed by staining with H2DCFDA. Both compounds led to an increase in ROS levels compared to the untreated control; however, the effect was significant only for **5** (Figure 5). These findings are consistent with cell cycle analysis, in which compound **5** exhibited a significant increase in the number of cells in the sub-G1 phase, indicative of apoptosis. Moreover, the elevated ROS levels can induce DNA damage, potentially leading to replication fork stalling and cell cycle arrest in the S phase, as observed for both aziridine phosphine oxides in the cell cycle analysis.

The aziridine phosphines and aziridine phosphine oxides presented herein underwent a preliminary investigation for their potential biological activity, marking the inaugural exploration of their properties. Initial assessments involved the determination of their antibacterial activity against reference strains of bacteria. However, all compounds demonstrated moderate efficacy only against *S. aureus*, with MIC values of 50 μM (Tables S1 and S2). Drawing conclusive insights into structure–activity relationships proves challenging due to this limited activity scope. The specificity for *S. aureus* may be attributed to the distinctive cell membrane structure of Gram-positive bacteria, potentially rendering it more permeable to these compounds compared to Gram-negative bacteria. Concerning clinical strains of *S. aureus*, only aziridine phosphine oxides **5**, **6**, and **7** exhibited antibacterial activity at the highest tested concentrations (MIC = 100 μM), which means that the presence of both phosphine oxide and a branched aliphatic substituent on the aziridine ring determines the antibacterial activity. Chiral phosphine oxides often exhibit increased steric hindrance compared to phosphines due to the presence of the oxygen atom. This can affect their interactions with biological targets or enzymes. The bulkier nature of chiral phosphine oxides can influence their binding affinities and selectivity. Moreover, chiral phosphine oxides possess an oxygen atom capable of hydrogen bonding, whereas phosphines lack this functionality. This enables chiral phosphine oxides to engage in additional interactions with biological targets or enzymes through hydrogen bonding, potentially influencing their biological activities [21]. The observed high MIC values and limited spectrum of antibacterial activity collectively affirm their unsuitability as promising candidates for antibacterial agents. Conversely, the compounds exhibited noteworthy anticancer activity, as evidenced by their capacity to inhibit cancer cell viability, with IC_50_ values comparable to the widely used anticancer drug cisplatin with moderate selectivity towards cancerous cells, signifying their potential as effective agents in the realm of anticancer therapeutics. 

Our comprehensive evaluation of all nine derivatives revealed that the phosphine oxide derivatives (**5**–**8**) exhibit enhanced activity against the tested cancer cells in comparison to the aziridine phosphines (**1**–**4**) (Table 1). This is consistent with the literature reports indicating that the presence of bulky substituents on the phosphorus atom enhances biological activity [18,22,23]. It was also confirmed that aziridine should have a protected amino group to exhibit cancer cell viability inhibition, indicated by a lack of compound **9** activity, and Carraminana et al. observed a similar effect [19]. The highest activity was achieved by aziridine derivatives that contained a branched aliphatic substituent in the aziridine ring, compounds **5** and **7** ((*S*)-isopropyl and (*S*)-isobutyl, respectively). The absolute configuration is also extremely important because compounds with the (*R*) configuration are much less active (compound **5** vs. **6**), which is in agreement with our previous work [14]. It is worth mentioning that aziridines with an (*S*) configuration are derived from naturally occurring amino acids also possessing an (*S*) configuration. 

Aziridine phosphine oxide derivatives caused viability inhibition of the tested cancer cells without causing damage to the cell membrane; nevertheless, they inhibited the cell cycle in the S phase (Figure 4), which strongly suggests that the cell viability decrease is due to antiproliferative effects. The mechanism of action elucidated for certain aziridine derivatives, such as mitomycin C and azinomycin (Figure 1), involves antitumor effects through DNA binding and subsequent inhibition of DNA replication. Mitomycin C, known as an antitumor agent, binds to the minor groove of DNA, while azinomycin B induces interstrand crosslinks, and both processes lead to the inhibition of DNA replication. Bearing in mind our results and the established role of aziridine derivatives as alkylating agents, it is hypothesized that our most potent compounds, **5** and **7**, may possess the potential to bind to DNA. Subsequent research efforts will involve targeted investigations, including BrdU proliferation tests and assessments of DNA binding activity through techniques such as molecular modeling simulations, comet assays for crosslink detection, DNA melting temperature studies, and DNA circular dichroism measurements to explore conformational changes in DNA morphology during interactions with these compounds [24,25,26]. 

While the antiproliferative activity of the tested aziridine phosphine oxides, **5** and **7**, evidenced by cell viability inhibition without concurrent cell membrane disruption measured by the LDH release assay, suggests a potential mechanism involving DNA interaction, it is noteworthy that at higher concentrations (≥50 μM), cell membrane destabilization was observed, indicative of cell lysis. This phenomenon may be attributed to elevated levels of reactive oxygen species (ROS) and apoptosis, the latter manifested by a subpopulation of cells in the sub-G1 phase of the cell cycle, observed even at lower concentrations corresponding to IC_50_ values (Figure 4 and Figure 5). This apoptotic effect aligns with our previous research on this class of compounds [15]. A similar mechanism in cancerous cells was observed for another aziridine derivative, imexon. Imexon is an antitumor agent that has selective activity in multiple myeloma but is also effective in the case of other cancer cell lines. Myeloma cells exposed to imexon showed classic morphologic features of apoptosis, increased levels of phosphatidylserine exposure and the accumulation of hydroxydeoxyguanosine (8-OHdG), an indicator of oxidative stress [27]. Pancreatic carcinoma cells treated with imexon showed a dose-dependent increase in ROS and caspase activation [28]. 

In summary, the specific cytotoxic mechanism underlying the effects of the tested aziridine derivatives, particularly the most active aziridine phosphine oxides, remains unclear and needs further in-depth investigation. Nevertheless, the present study underscores their considerable potential as anticancer agents.

## 3. Materials and Methods

*Chemistry:* All aziridine phosphines (**1**–**4**) and aziridine phosphine oxides (**5**–**9**) were prepared and purified according to previously described procedures [16,17,29]. The synthetic procedures are presented in the Supplementary Materials.

Antibacterial activity: The in vitro antibacterial activity of nine tested aziridine phosphines (**1**–**4**) and aziridine phosphine oxides (**5**–**9**) was evaluated against reference bacterial strains (*E. coli* NCTC 8196, *S. aureus* ATCC 6538, *K. pneumoniae* ATCC 13883, *P. aeruginosa* NCTC 6749) and 12 *S. aureus* clinical isolates, including two methicillin-resistant isolates (MRSA, D15 and D17) obtained from infected patients hospitalized at the Oncological Hospital in Lodz (Poland). All strains were kept frozen at −80 °C in Tryptic Soy Broth with 15% glycerol. The minimal inhibitory concentration (MIC) is defined as the lowest concentration of the compound that prevents visible growth of bacteria using the microdilution method followed the EUCAST guidelines [30]. Each compound was added to 96-well microplates in two-fold dilutions at concentrations from 3.125 to 100 μM in Mueller–Hinton broth. All compounds were dissolved in DMSO, and its final concentration did not exceed 1% and had no effects on bacterial growth. Bacterial culture was added into wells at a density of 5 × 10^5^ CFU mL^−1^, and plates were incubated at 37 °C for 18 h. As a positive control, an untreated bacterial culture was used, and a clear culture medium served as a negative control. The optical density at OD_600_ was measured using the SpectraMax i3 Multi-Mode Platform (Molecular Devices, San Jose, CA, USA). Ciprofloxacin hydrochloride (CIP, Sigma-Aldrich, Darmstadt, Germany) was used as a reference antimicrobial drug.

Cell viability inhibition: The in vitro effects on cell viability were determined using the 3-(4,5-dimethylthiazol-2-yl)-2,5-diphenyltetrazolium bromide (MTT) assay on non-tumorigenic murine fibroblasts L929 (ATTC^®^—CCL-1) as well as two cancer cell lines: human cervical epithelioid carcinoma HeLa cells (ATTC^®^—CCL-2™) and endometrial adenocarcinoma Ishikawa cells (ECACC 99040201). Cells were cultured on 96-well microplates at a density of 1 × 10^4^ cells/well following the international [31] in Dulbecco’s Modified Eagle’s Medium (DMEM, Biowest) supplemented with 10% fetal bovine serum (FBS, Biowest, Nuaillé, France), 100 U/mL of penicillin, and 100 μg/mL of streptomycin (Biowest, Nuaillé, France). After overnight incubation at 37 °C and 5% CO_2_, the growth medium was removed and 100 μL of fresh media with two-fold dilutions of the tested compounds at concentrations of 3.125–100 μM were added. All compounds were dissolved in DMSO, the final concentration of which did not exceed 1% and had no influence on cell culture growth. Untreated cells in medium were used as a positive control, while a clear culture medium served as a negative control. After 24 h of incubation, the medium was removed and 3-(4,5-dimethylthiazol-2-yl)-2,5-diphenyltetrazolium bromide (MTT, Sigma-Aldrich (Darmstadt, Germany) at a concentration of 5 mg/mL was added. Plates were incubated for the next 2 h at 37 °C and 5% CO_2_. Formazan crystals were solubilized in 150 μL of DMSO and absorbance was measured at 570 nm using the SpectraMax i3 Multi-Mode Platform (Molecular Devices, San Jose, CA, USA). The results of three independent experiments were presented as mean arithmetic values and the percentage of viability inhibition was calculated, considering the untreated control as having 100% viability. The IC_50_ values of the compounds (the drug concentration that inhibits cell viability by 50%) were calculated with the GraphPad Prism 7 software using nonlinear regression.

LDH release assay: The activity of **5** and **7** on cell membrane destabilization was determined with the CytoTox 96^®^Non-Radioactive Cytotoxicity Assay (Promega, Madison, WI, USA). HeLa cells (ATTC Catalog No. CCL-2^TM^) were plated in 96-well microplates at a density of 1 × 10^4^ cells/well in Dulbecco’s Modified Eagle’s Medium F12 (DMEM-F12, Biowest, Nuaillé, France) supplemented with 10% fetal bovine serum (FBS, Biowest, Nuaillé, France), 100 units/mL of penicillin G, and 100 μg/mL of streptomycin (Biowest, Nuaillé, France). After overnight incubation at 37 °C and 5% CO_2_, the culture medium was replaced with a fresh medium containing compounds at concentrations ranging from 3.125 to 50 μM. Additionally, the negative control (wells filled only with a medium), positive control (untreated cells), and maximum LDH release control (100% lysed cells) were included in the experiment. The maximum LDH release control was prepared by a 45 min treatment of cells with 1× lysis solution. Cells were incubated with compounds at 37 °C and 5% CO_2_ for 24 h. After incubation, 50 μL of supernatant from each well was transferred to a new 96-well plate and CytoTox 96^®^ Reagent (50 µL) was added. The plate was then covered with foil and incubated for 30 min at room temperature. After incubation, Stop Solution (50 μL) was added to each well, and the absorbance was measured at 490 nm using the SpectraMax i3 Multi-Mode Platform (Molecular Devices, San Jose, CA, USA). The results were shown as a percentage of LDH release by dividing the experimental LDH release × 100% by the maximum LDH release control from two independent experiments.

Cell cycle analysis: The distribution of cell cycle phases was measured using flow cytometry and propidium iodide (PI) staining. The PI intercalates into the major groove of double-stranded DNA, producing a highly fluorescent signal when excited at 488 nm, with a broad emission centered around 600 nm. PI binds in proportion to the amount of DNA present in the cell. HeLa cells were seeded on 60 mm dishes at a density of 5 × 10^5^ in 3 mL of Dulbecco’s Modified Eagle’s Medium F12 (DMEM-F12, Biowest, Nuaillé, France) supplemented with 10% fetal bovine serum (FBS, Biowest), 100 units/mL of penicillin G and 100 μg/mL of streptomycin (Biowest, Nuaillé, France) and incubated for 24 h at 37 °C and 5% CO_2_. Then, compounds **5** or **7** were added at a concentration corresponding to their IC_50_ values and the plate was incubated for the next 24 h. Untreated cells were used as a positive control. The cells were then harvested by trypsinization, suspended in culture medium (1 mL), centrifuged at 3600 rpm for 10 min, and washed twice with dPBS (Dulbecco’s phosphate-buffered saline, Cytogen–Polska Sp. z o.o, Zgierz, Poland). The cell sediment was resuspended in dPBS (600 μL) and fixed in 70% ice-cold ethanol. After storing the cells at 4 °C for 24 h, they were stained in PI staining solution (600 µL, 50 μg/mL of PI, and 100 μg/mL of RNAse A in dPBS) and incubated for 30 min at 37 °C in darkness. The cell cycle distribution was analyzed by flow cytometry using an LSRII (Becton Dickinson, Franklin Lakes, NJ, USA) instrument at 488 nm excitation and 535/617 emission wavelengths, and the cell cycle phase distribution from two independent experiments (10,000 cell counts/sample) was determined with the FlowJo 7.6.1 software (FlowJo, LLC, Ashland, OR, USA) using a built-in cell cycle analysis module (Watson pragmatic algorithm). 

Reactive oxygen species measurement: The intracellular level of ROS was analysed by staining with 2′,7′–dichlorofluorescein diacetate (H2DCFDA, Sigma-Aldrich, Darmstadt, Germany). This cell-permeable probe is deacetylated by cellular esterases to a non-fluorescent compound, which is later oxidized by ROS into 2′,7′–dichlorofluorescein (DCF). This product is highly fluorescent and can be detected on a flow cytometer. HeLa cells were seeded on 60 mm dishes at a density of 5 × 10^5^ in 3 mL of Dulbecco’s Modified Eagle’s Medium F12 (DMEM-F12, Biowest, Nuaillé, France) supplemented with 10% fetal bovine serum (FBS, Biowest), 100 units/mL of penicillin G, and 100 μg/mL of streptomycin (Biowest, Nuaillé, France) and incubated for 24 h at 37 °C and 5% CO_2_. Compounds **5** or **7** were added at a concentration corresponding to their IC_50_ values and the plate was incubated for 24 h. Untreated cells were used as a positive control. The cells were then harvested by trypsinization, suspended in culture medium (1 mL), centrifuged at 3600 rpm for 10 min, and washed twice with dPBS (Dulbecco’s phosphate-buffered saline, Cytogen—Polska Sp. z o.o, Zgierz, Poland). The cell sediment was resuspended in dPBS to obtain the same cell density, centrifuged and resuspended in H2DCFDA solution in dPBS (5 µM, 600 µL), and incubated for the next 30 min at 37 °C in darkness. The level of intracellular ROS generation was analysed by flow cytometry using an LSRII (Becton Dickinson, Franklin Lakes, NJ, USA) instrument at 490 nm excitation and 525 nm emission wavelengths, and the ROS levels from two independent experiments (10,000 cell counts/sample) was determined with the FlowJo 7.6.1 software (FlowJo, LLC, Ashland, OR, USA).

Statistical analysis: An analysis of statistical significance was performed with the Prism GraphPad 7 software using a two-way analysis of variance (ANOVA) followed by a Bonferroni multiple comparison test. For statistical analysis, significance levels of *p* < 0.05 (*) were considered. The results were presented as mean ± SD from at least two independent experiments.

## 4. Conclusions

In conclusion, we present the synthesis of chiral aziridine phosphines and aziridine phosphine oxides, demonstrating their antiproliferative activity against cancer cells. Following the evaluation of the biological activity of all nine derivatives, it was found that phosphine oxide derivatives exhibit greater activity against the tested cells, both bacterial and cancerous, compared to analogous aziridine phosphines. The highest activity was observed in aziridine derivatives **5**, **6**, and **7**, containing a branched aliphatic substituent in the aziridine ring. Furthermore, aziridine phosphine oxide derivatives demonstrated a cell viability inhibition on the tested cancer cells at concentrations that did not induce damage to the cell membrane. The antiproliferative effect of **5** and **7** can be explained by the cell cycle arrest in the S phase, resulting in the inhibition of replication. Additionally, an apoptotic effect of these compounds was observed, which is also consistent with our previous research on this group of compounds [15]. Both effects on cancer cells may be attributed to the generation of reactive oxygen species by the tested aziridine derivatives. The findings presented in this manuscript constitute preliminary investigations wherein we conducted screening in vitro tests for these compounds for the first time to elucidate potential biological activities. The results obtained have their limitations, and future research endeavors are planned to broaden the scope of our investigations by incorporating a number of both cancerous and normal cell lines and specific experiments to elucidate the mechanisms of action underlying the enhanced efficacy exhibited by the most promising compounds on cancerous cells.

## Figures and Tables

**Table 1:** Effect of aziridine phosphines (**1**–**4**) and aziridine phosphine oxides (**5**–**9**) on the viability of L929, HeLa, and Ishikawa cells after 24 h treatment. The results are expressed as IC_50_ ± SD from three independent experiments and the selectivity index (SI). An SI > 1.0 indicates a compound with greater efficacy of cell viability inhibition against HeLa or Ishikawa cells than against normal fibroblasts L929. CisPt—cisplatin, nd—not possible to determine. The most active compounds are highlighted in gray background.

		IC_50_ (µM)		
		L929	HeLa	Ishikawa
Aziridine phosphines	**1**	68.1 ± 11.2	>100 (SI < 1)	13.9 ± 7.1 (SI = 4.9)
	**2**	>100	>100 (SI–nd)	14.8 ± 6.5 (SI > 6.7)
	**3**	>100	30.3 ± 4.5 (SI > 3.3)	>100 (SI–nd)
	**4**	22 ± 3.4	39.2 ± 6.7 (SI < 1)	>100 (SI < 1)
Aziridine phosphine oxides	**5**	**10.6 ± 3.2**	**6.4 ± 2.1** **(SI = 1.7)**	**4.6 ± 2.3** **(SI = 2.3)**
	**6**	13.7 ± 3.1	40.7 ± 11.1 (SI < 1)	16.6 ± 2.5 (SI < 1)
	**7**	**17.5 ± 2.1**	**7.1 ± 2.6** **(SI = 2.5)**	**10.5 ± 3.1** **(SI = 1.7)**
	**8**	17.0 ± 4.4	19.6 ± 3.2 (SI < 1)	23.4 ± 7.6 (SI < 1)
	**9**	>100	48.8 ± 12.3 (SI > 2.0)	>100 (SI–nd)
	**CisPt**	26.2 ± 7.5	10.4 ± 4.4 (SI > 2.5)	17.3 ± 5.2 (SI = 1.5)

## Data Availability

The original contributions presented in the study are included in the article and Supplementary Material, further inquiries can be directed to the corresponding authors.

## Supplementary Materials

The following supporting information can be downloaded at: https://www.mdpi.com/article/10.3390/molecules29071430/s1. Scheme 1. Reduction of aziridinylphoshine oxides to form phosphines **1**–**4**. Scheme 2. Synthesis of chiral aziridine phosphine oxides **5**–**8**. Scheme 3. Synthesis of aziridinephosphine oxide **9**. Table S1. In vitro antibacterial activity of aziridine phosphines (**1**–**4**) and aziridine phosphine oxides (**5**–**9**) against reference bacteria, expressed as a minimal inhibitory concentration (MIC, µM). CIP—ciprofloxacin. Table S2. In vitro antibacterial activity of aziridine phosphines (**1**–**4**) and aziridine phosphine oxides (**5**–**9**) against clinical strains of *S. aureus* expressed as a minimal inhibitory concentration (MIC, µM).
