# Supplementary material for: A Study on the Biological Activity of Optically Pure Aziridine Phosphines and Phosphine Oxides
Source: Molecules. 2024 Mar 22;29(7):1430. doi: 10.3390/molecules29071430 (PMC11012799; doi:10.3390/molecules29071430)
Supplement: Supplementary file 1 [file molecules-29-01430-s001.zip › molecules-2878589-supplementary.pdf]

Supplementary Material

A Study on the Biological Activity of Optically Pure Aziridine Phosphines and Phosphine Oxides

Aleksandra Kowalczyk [1,*], Adam M. Pieczonka [2,*], Hassan Kassassir [3], Michał Rachwalski [2] and Paweł Stączek [1]

[1] Department of Molecular Microbiology, Faculty of Biology and Environmental Protection, University of Lodz, Banacha 12/16, 90-237 Lodz, Poland; pawel.staczek@biol.uni.lodz.pl
[2] Department of Organic and Applied Chemistry, Faculty of Chemistry, University of Lodz, Tamka 12, 91-403 Lodz, Poland; michal.rachwalski@chemia.uni.lodz.pl
[3] Cellular Signalling Laboratory, Institute for Medical Biology, Polish Academy of Sciences, Lodowa 106, 93-232 Lodz, Poland; hkassassir@cbm.pan.pl
* Correspondence: aleksandra.strzelczyk@biol.uni.lodz.pl (A.K.); adam.pieczonka@chemia.uni.lodz.pl (A.M.P.)

Table of content:

1. *Chemistry*
2. *Antibacterial activity*

1. Chemistry

Aziridine phosphines **1-4** were obtained *via* triethoxysilane and titanium (IV) isopropoxide-mediated reduction of the corresponding aziridine phosphine oxides **5-8** [17] (Scheme 1).

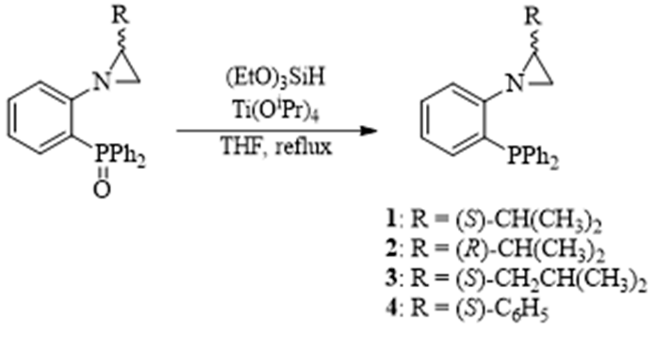

Scheme 1. Reduction of aziridinylphoshine oxides to form phosphines **1-4**.

Reduction of Phosphinoyl-Aziridines to Aziridine-Phosphines **1–4**—General Procedure:
To a stirred mixture of phosphine oxide (1 mmol) and triethoxysilane (3 mmol) in dry THF (5 mL), titanium isopropoxide (0.1 mmol) was added. The reaction mixture was refluxed until the completion of the reaction (TLC monitored) and cooled to room temperature. A solution of NaOH (1 M, 10 mL) was added. The resulting mixture was stirred for 2 h at room temperature and then extracted with ethyl acetate (4 × 15 mL). The organic layer was dried with $MgSO_4$ and evaporated in vacuo. The crude product was purified via column chromatography on silica gel (hexane:ethyl acetate 7:1).

(2S)-1-[2-(Diphenylphosphinophenyl]-2-isopropylaziridine **1:**

Colorless oil, 65% yield; [α]D20 = +21.3 (c 0.5, CHCl3);

^{1}H NMR (600 MHz, CDCl3): δ = 0.87 (d, J = 6.7 Hz, 3H, CH3), 1.06 (d, J = 6.7 Hz, 3H, CH3), 1.71–1.75 (m, 1H) 1.77–1.80 (m, 1H), 2.07–2.11 (m, 2H), 6.76–6.80 (m, 1H, CHar), 6.88–6.91 (m, 1H, CHar), 6.93–6.97 (m, 1H, CHar), 7.23–7.27 (m, 1H, CHar), 7.28–7.31 (m, 2H, CHar), 7.31–7.34 (m, 3H, CHar), 7.36–7.41 (m, 5H, CHar) ppm.

^{13}C NMR (150 MHz, CDCl3): δ = 18.5 (CH3), 20.8 (CH3), 30.1 (CH), 34.8 (d, J = 6.8 Hz, CH2N), 46.3 (d, J = 5.5 Hz, CHN), 119.4 (d, J = 3.2 Hz, CHar), 122.3 (CHar), 128.3 (2 CHar), 128.4 (CHar), 128.7 (CHar), 129.6 (2 CHar), 133.8 (CHar), 133.9 (CHar), 134.2 (CHar), 134.4 (CHar), 137.0 (CHar), 137.1 (CHar), 137.2 (CHar), 137.3 (CHar), 157.7, 157.8 (2 Cq ar) ppm.

^{31}P NMR (243 MHz, CDCl3): δ = –17.3 ppm.

Anal. Calcd. for $C_{23}H_{24}NP$ C, 80.00; H, 7.00; N, 4.10; Found: C, 79.95; H, 6.98; N, 4.12.

(2*R*)-1-[2-(Diphenylphosphinophenyl]-2-isopropylaziridine **2**:

Colorless oil, 60% yield; [*α*]D20 = -60.2 (c 0.5, CHCl3);

^{1}H NMR (600 MHz, CDCl3): δ = 0.87 (d, J = 6.7 Hz, 3H, CH3), 1.05 (d, J = 6.7 Hz, 3H, CH3), 1.72–1.77 (m, 1H), 1.77–1.80 (m, 1H), 2.08–2.11 (m, 2H), 6.76–6.80 (m, 1H, CHar), 6.88–6.91 (m, 1H, CHar), 6.93–6.97 (m, 1H, CHar), 7.24–7.27 (m, 1H, CHar), 7.27–7.31 (m, 2H, CHar), 7.31–7.34 (m, 3H, CHar), 7.35–7.40 (m, 5H, CHar) ppm.

^{13}C NMR (150 MHz, CDCl3): δ = 18.5 (CH3), 20.7 (CH3), 30.1 (d, J = 1.8 Hz, CH), 34.6 (d, J = 6.8 Hz, CH2N), 46.3 (d, J = 5.5 Hz, CHN), 119.4 (d, J = 3.4 Hz, CHar), 122.3 (CHar), 128.2 (CHar), 128.4 (CHar), 128.5 (CHar), 128.7 (CHar), 129.6 (CHar), 133.8 (CHar), 133.9 (CHar), 134.2 (CHar), 134.3 (CHar), 134.4 (CHar), 137.0 (CHar), 137.1 (CHar), 137.2 (CHar), 137.3 (CHar), 157.7, 157.8 (2 Cq ar) ppm.

^{31}P NMR (243 MHz, CDCl3): δ = –17.3 ppm

Anal. Calcd. for $C_{23}H_{24}NP$ C, 80.00; H, 7.00; N, 4.10; Found: C, 80.05; H, 7.21; N, 4.11.

(2*S*)-1-[2-(Diphenylphosphinophenyl]-2-isobutylaziridine **3**:

Colorless oil, 58% yield; [*α*]D20 = +32.6 (c 0.5, CHCl3);

^{1}H NMR (600 MHz, CDCl3): δ = 0.88 (d, J = 6.7 Hz, 3H, CH3), 0.92 (d, J = 6.7 Hz, 3H, CH3), 1.07–1.12 (m, 1H, CH(CH3)2), 1.62–1.70 (m, 1H), 1.88–1.94 (m, 1H), 2.04 (m, 2H), 2.09–2.14 (m, 1H), 6.78–6.81 (m, 1H, CHar), 6.89–6.95 (m, 2H, CHar), 7.25–7.28 (m, 1H, CHar), 7.29–7.32 (m, 2H, CHar), 7.32–7.38 (m, 8H, CHar) ppm.

^{13}C NMR (150 MHz, CDCl3): δ = 22.6 (CH3), 22.7 (CH3), 26.9 (CH), 35.7 (d, J = 5.5 Hz, CH2), 39.7 (d, J = 4.7 Hz, CH2N), 41.4 (d, J = 3.3 Hz, CHN), 119.6 (CHar), 122.4 (CHar), 128.3 (CHar), 128.4 (2 CHar), 128.5 (CHar), 128.6 (CHar), 129.7 (2 CHar), 129.8 (CHar), 133.8 (CHar), 134.0 (CHar), 134.1 (CHar), 134.4 (CHar), 137.1 (CHar), 137.3 (CHar), 154.7 (Cq ar), 157.6

(Cq ar) ppm.

^{31}P NMR (243 MHz, CDCl3): δ = – 17.3 ppm.

Anal. Calcd. for $C_{24}H_{26}NP$ C, 80.20; H, 7.30; N, 3.90; Found: C, 80.37; H, 7.14; N, 3.74.

(2*S*)-1-[2-(Diphenylphosphinophenyl]-2-phenylaziridine **4**:

Colorless oil, 56% yield; [*α*]D20 = +25.8 (c 0.5, CHCl3);

^{1}H NMR (600 MHz, CDCl3): δ = 2.32 (d, J = 3.2 Hz, 1H, CHN), 2.54 (d, J = 6.4 Hz, 1H, CHN), 3.19 (dd, J = 3.2 Hz, 6.3 Hz, 1H, CHN), 6.75–6.79 (m, 1H, CHar), 6.93–6.97 (m, 1H, CHar), 7.00–7.04 (m, 1H, CHar), 7.08–7.12 (m, 2H, CHar), 7.16–7.20 (m, 2H, CHar), 7.21–7.25 (m, 5H, CHar), 7.28–7.37 (m, 6H, CHar), 7.39 (s, 1H, CHar) ppm.

^{13}C NMR (150 MHz, CDCl3): δ = 39.2 (d, J = 5.6 Hz, CH2N), 42.5 (d, J = 6.6 Hz, CHN), 119.2 (CHar), 119.3 (CHar), 122.7 (CHar), 126.1 (3 CHar), 126.9 (CHar), 128.0 (CHar), 128.2 (CHar), 128.3 (CHar), 128.4 (CHar), 128.5 (CHar), 129.5 (2 CHar), 133.8 (CHar), 134.0 (CHar), 134.2 (CHar), 134.3 (CHar), 136.3 (CHar), 136.4 (CHar), 136.6 (CHar), 139.1 (CHar), 156.0, 156.1 (2 Cq ar) ppm.

^{31}P NMR (243 MHz, CDCl3): δ = – 15.9 ppm.

Anal. Calcd. for $C_{26}H_{22}NP$: C, 82.30; H, 5.80; N, 3.70; Found: C, 82.26; H, 5.83; N, 3.67.

In turn, aziridine phosphine oxides **5-8** were synthesized as previously described [18] from *o*-bromoanisole, diphenylphosphinic acid and the corresponding enantiomerically pure aziridines (Scheme 2).

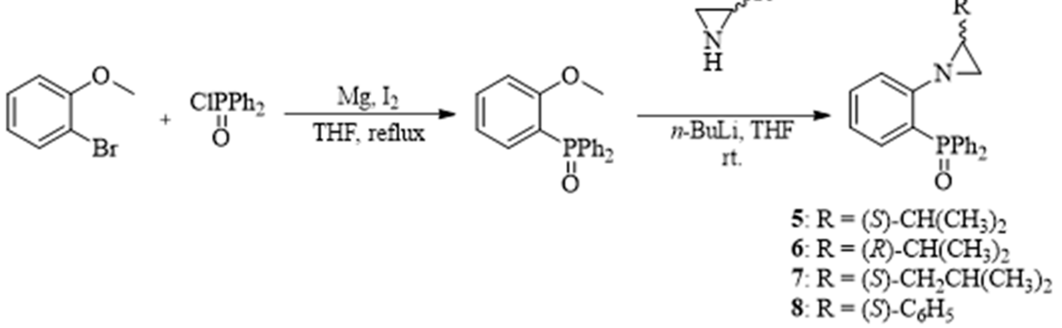

Scheme 2. Synthesis of chiral aziridine phosphine oxides **5-8**.

General procedure of the synthesis of compounds **5-8**.

To the solution of the corresponding aziridine 5 (1.50 mmol) in anhydrous THF (2 mL) was added dropwise n-BuLi (0.82 mL, 1.6 mmol, 1.95 M solution in hexane) at -79 °C under argon. The mixture was stirred at room temperature for 2 h under argon and phosphine oxide (0.456 g, 1.48 mmol) was added at 0 °C. The stirring was continued overnight at room temperature. Next, the mixture was diluted with ether and quenched with saturated aqueous solution of ammonium chloride. The organic phase was washed with brine, dried over anhydrous MgSO4 and concentrated in vacuo. The residue was purified via column chromatography (silica gel, hexane/ethyl acetate/methanol 3:3:0.25) to afford the compounds **5-8**.

(2*S*)-1-[2-(diphenylphosphinoyl)phenyl]-2-isopropylaziridine **5**:

Yellowish oil (0.42 g, 59%); $[\alpha]D_{20}$ = +23.2 (c 0.5, $CHCl_3$);

^{1}H NMR (600 MHz, CDCl3): δ = 0.69 (d, J = 6.8 Hz, 3H, CH3), 0.85 (d, J = 6.8 Hz, 3H, CH3), 1.57–1.62 (m, 1H, CHN), 1.82 (d, J = 3.7 Hz, 1H, CHN), 2.07 (d, J = 6.8 Hz, 1H, CHN), 2.20–2.23 (m, 1H, CH(CH3)2), 6.92–6.98 (m, 2H, CHar), 7.13–7.17 (m, 1H, CHar), 7.40–7.50 (m, 6H, CHar), 7.51–7.56 (m, 1H, CHar), 7.75–7.79 (m, 2H, CHar), 7.82–7.85 (m, 2H, CHar) ppm.

^{13}C NMR (150 MHz, CDCl3): δ = 18.3 (CH3), 19.8 (CH3), 29.6 (CHN), 36.1 (CHN), 45.7 (CH(CH3)2), 120.1 (d, J = 7.7 Hz, CHar), 121.5 (d, J = 12.0 Hz, CHar), 124.3, 124.9 (2 CHar), 128.1 (d, J = 12.0 Hz, CHar), 128.3 (d, J = 12.0 Hz, CHar), 131.1 (d, J = 3.2 Hz, CHar), 131.4 (d, J = 3.2 Hz, CHar), 131.6 (d, J = 9.8 Hz, CHar), 132.1 (CHar), 132.2 (CHar), 133.2 (CHar), 133.6 (CHar), 133.8 (CHar), 134.2 (CHar), 134.5 (CHar), 134.8 (d, J = 9.8 Hz, CHar), 158.1 (d, J = 4.6 Hz, CHar) ppm.

^{31}P NMR (243 MHz, CDCl3): δ = 28.7 ppm.

Anal. calcd. for $C_{23}H_{24}NOP$: C 76.45, H 6.65, N 3.88; found: C 76.53, H 6.32, N 3.75.

(2*R*)-1-[2-(diphenylphosphinoyl)phenyl]-2-isopropylaziridine **6**:

Yellowish oil (0.51 g, 71%); $[\alpha]D_{20}$ = -9.6 (c 0.5, $CHCl_3$);

^{1}H NMR (600 MHz, CDCl3): δ = 0.69 (d, J = 6.8 Hz, 3H, CH3), 0.85 (d, J = 6.8 Hz, 3H, CH3), 1.57–1.61 (m, 1H, CHN), 1.81 (d, J = 3.7 Hz, 1H, CHN), 2.07 (d, J = 6.8 Hz, 1H, CHN), 2.20–2.23 (m, 1H, CH(CH3)2), 6.92–6.98 (m, 2H, CHar), 7.10–7.17 (m, 1H, CHar), 7.39–7.51 (m, 6H, CHar), 7.51–7.58 (m, 1H, CHar), 7.72–7.79 (m, 2H, CHar), 7.81–7.86 (m, 2H, CHar) ppm.

^{13}C NMR (150 MHz, CDCl3): δ = 18.3 (CH3), 19.8 (CH3), 29.6 (CHN), 31.6 (CHN), 45.7 (CH(CH3)2), 120.1 (d, J = 7.7 Hz, CHar), 121.5 (d, J = 12.0 Hz, CHar), 124.3, 124.9 (2 CHar), 128.1 (d, J = 12.0 Hz, CHar), 128.3 (d, J = 12.0 Hz, CHar), 133.1 (d, J = 3.2 Hz, CHar), 131.4 (d, J = 3.2 Hz, CHar), 131.5 (d, J = 9.8 Hz, CHar), 131.6 (d, J = 9.8 Hz, CHar), 132.1 (d, J = 9.8 Hz, CHar), 133.2 (CHar), 133.5 (CHar), 133.7 (CHar), 134.2 (CHar), 134.7 (CHar), 134.8 (d, J = 9.8 Hz, CHar), 158.1 (d, J = 4.6 Hz, CHar) ppm.

^{31}P NMR (243 MHz, CDCl3): δ = 28.8 ppm.

Anal. calcd. for $C_{23}H_{24}NOP$: C 76.45, H 6.65, N 3.88; found: C 76.57, H 6.38, N 3.78.

(2*S*)-1-[2-(diphenylphosphinoyl)phenyl]-2-isobutylaziridine **7**:

Yellowish oil (0.43 g, 57%); $[\alpha]D_{20}$ = +24.9 (c 0.5, $CHCl_3$);

^{1}H NMR (600 MHz, CDCl3): δ = 0.87 (d, J = 6.7 Hz, 3H, CH3), 0.89 (d, J = 6.7 Hz, 3H, CH3), 0.91–0.97 (m, 1H, CH(CH3)2), 1.49–1.54 (m, 1H, CHN), 1.60–1.67 (m, 1H, CH-2CH(CH3)2), 1.76 (d, J = 3.7 Hz, 1H, CHN), 2.13 (d, J = 6.2 Hz, 1H, CH¬2CH(CH3)2), 2.33–2.37 (m, 1H, CHN), 6.94–6.96 (m, 2H, CHar), 7.16–7.19 (m, 1H, CHar), 7.41–7.49 (m, 5H, CHar), 7.50–7.55 (m, 2H, CHar), 7.73–7.77 (m, 2H, CHar), 7.79–7.83 (m, 2H, CHar) ppm.

^{13}C NMR (150 MHz, CDCl3): δ = 22.7 (CH3), 22.9 (CH3), 27.0 (CH(CH3)2), 37.8 (CHN), 39.6 (CHN), 41.5 (CH2CH(CH3)2), 120.4 (d, J = 8.5 Hz, CHar), 121.7 (d, J = 12.0 Hz, CHar), 124.7 (CHar), 125.4 (d, J = 7.7 Hz, CHar), 128.2 (d, J = 6.1 Hz, CHar), 128.3 (d, J = 12.0 Hz, CHar), 131.3 (d, J = 2.1 Hz, CHar), 131.4 (d, J = 3.2 Hz, CHar), 131.7 (d, J = 9.8 Hz, CHar), 132.1 (CHar), 132.2 (CHar), 132.9 (CHar), 133.2 (d, J = 2.1 Hz, CHar), 133.6 (CHar), 133.7 (CHar), 134.4 (CHar), 134.7 (d, J = 9.8 Hz, CHar), 157.8 (d, J = 4.6 Hz, CHar) ppm.

^{31}P NMR (243 MHz, CDCl3): δ = 31.4 ppm.

Anal. calcd. for $C_{24}H_{26}NOP$: C 76.80, H 6.93, N 3.73; found: C 76.71, H 6.97, N 3.78.

(2*S*)-1-[2-(diphenylphosphinoyl)phenyl]-2-phenylaziridine **8**:

Yellowish oil (0.53 g, 67%); $[\alpha]D_{20}$ = +58.1 (c 0.5, $CHCl_3$);

^{1}H NMR (600 MHz, CDCl3): δ = 2.01 (d, J = 3.5 Hz, 1H, CHN), 2.58 (d, J = 6.5 Hz, 1H, CHN), 3.34–3.36 (m, 1H, CHN), 6.75–6.87 (4m, 5H, CHar), 6.95–6.96 (m, 3H, CHar), 7.06–7.28 (3m, 7H, CHar), 7.37–7.38 (2m, 4H, CHar) ppm.

^{13}C NMR (150 MHz, CDCl3): δ = 41.6 (CHN), 42.4 (CHN), 120.5 (3 CHar), 122.1, 122.2 (2 CHar), 124.7 (CHar),125.4 (4 CHar), 126.0 (CHar), 126.9 (d, J = 4.3 Hz, CHar), 128.2 (d, J = 3.4 Hz, 2 CHar), 128.4 (d, J = 3.4 Hz, CHar), 131.3 (d, J = 2.3 Hz, CHar), 131.4 (d, J = 9.7 Hz, 2 CHar), 131.7 (d, J = 8.7 Hz, CHar), 131.9 (d, J = 9.7 Hz, CHar), 133.4 (d, J = 9.7 Hz, CHar), 134.3 (CHar), 135.0 (CHar), 156.8 (d, J = 4.3 Hz, CHar) ppm.

^{31}P NMR (243 MHz, CDCl3): δ = 35.5 ppm.

Anal. calcd. for $C_{26}H_{22}NOP$: C 78.99, H 5.57, N 3.54; found: C 79.03, H 5.61, N 3.58.

Finally, aziridine phosphine oxide **9** containing free N-H group at aziridine ring was prepared according to our previous protocol [22] from (*S*)-2-phenylaziridine and diphenylphosphinic chloride in the presence of *sec*-BuLi (Scheme 3).

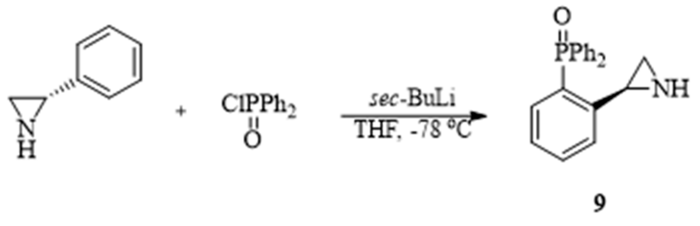

9

Scheme 3. Synthesis of aziridinephosphine oxide **9**.

Synthesis of (2*S*)-2-diphenylphosphinoyl-2-phenylaziridine **9**:

Sec-BuLi (1.43 mL, 2 mmol, 1.4 M solution in cyclohexane) was added dropwise to a solution of (*S*)-2-phenylaziridine (119 mg, 1 mmol) in anhydrous tetrahydrofurane (10 mL) at -78 °C under argon. The resulting brown solution was stirred at this temperature for 2 h and diphenylphosphinic chloride (237 mg, 1 mmol) was added. The mixture was stirred at -78 °C

for 2 h and then warmed up to room temperature. The reaction was quenched by the addition of saturated aqueous NH_4Cl (5 mL). The mixture was poured into water and extracted with Et2O (3 x 10 mL). The combined organic layers were dried over Na2SO4 and concentrated in vacuo. The crude product was purified via column chromatography on silica gel (hexane:ethyl acetate 50:50).

(2*S*)-2-diphenylphosphinoyl-2-phenylaziridine **9**:

Slightly yellow solid (0.22 g, 69%); $[\alpha]D_{20}$ = +18.0 (c 0.5, $CHCl_3$);

^{1}H NMR (600 MHz, $CDCl_3$): δ = 1.84 (br. s, 1H, NH), 2.24 (dd, J = 2.0 Hz, 13.0 Hz, 1H, CHN), 2.95 (dd, J = 6.0 Hz, 17.9 Hz, 1H, CHN), 3.78 (dq, J = 3.2 Hz, 6.0 Hz, 15.6 Hz, 1H, CHN), 7.30–7.33 (m, 1H, CHar), 7.35–7.40 (m, 5H, CHar), 7.44–7.48 (m, 1H, CHar), 7.49–7.53 (m, 2H, CHar), 7.54–7.57 (m, 1H, CHar), 7.87–7.92 (m, 2H, CHar), 7.98–8.03 (m, 2H, CHar) ppm.

^{13}C NMR (150 MHz, $CDCl_3$): δ = 33.0 (d, J = 7.4 Hz, CH2N), 37.0 (d, J = 5.0 Hz, CHN), 126.2 (CHar), 127.8 (CHar), 128.4 (CHar), 128.5 (2 CHar), 128.6 (CHar), 131.5 (CHar), 131.6 (2 CHar), 131.7 (CHar), 131.9 (d, J = 3.2 Hz, CHar), 132.0 (d, J = 2.1 Hz, CHar), 132.1 (CHar), 132.4 (CHar), 132.9 (CHar), 133.3 (CHar), 137.6, 137.7 (2 Cq ar) ppm.

^{31}P NMR (243 MHz, $CDCl_3$): δ = 32.9 ppm.

Anal. Calcd. for $C_{20}H_{18}NOP$: C 75.20, H 5.70, N 4.40; found C 75.28, H 5.89, N 4.60.

2. Antibacterial activity

Table S1. In vitro antibacterial activity of aziridine phosphines (**1**-4**)** and aziridine phosphine oxides (**5**-9**)** against reference bacteria, expressed as a minimal inhibitory concentration (MIC, μM). CIP – ciprofloxacin.

	MIC (μM)									
	Aziridine phosphines				Aziridine phosphine oxides					
	**1**	**2**	**3**	**4**	**5**	**6**	**7**	**8**	**9**	**CIP**
Escherichia coli NCTC 8196	>100	>100	>100	>100	>100	>100	>100	>100	>100	0.04
Staphylococcus aureus ATCC 6538	**50**	**50**	**50**	**50**	**50**	**50**	**50**	**50**	**50**	1.6
Klebsiella pneumoniae ATCC 13883	>100	>100	>100	>100	>100	>100	>100	>100	>100	0.2

Pseudomonas aeruginosa NCTC 6749	>100	>100	>100	>100	>100	>100	>100	>100	>100	2.5

Table S2. In vitro antibacterial activity of aziridine phosphines (**1-4**) and aziridine phosphine oxides (**5-9**) against clinical strains of *S. aureus* expressed as a minimal inhibitory concentration (MIC, μM).

	**MIC (μM)**								
	Aziridine phosphines				Aziridine phosphine oxides				
	**1**	**2**	**3**	**4**	**5**	**6**	**7**	**8**	**9**
S. aureus	**naso-pharynx isolates**								
C4	>100					**100**		>100	
C7						**100**			
C8						**100**			
C19						**100**			
	**ulcers/furunlcers isolates**								
D12	>100					**100**		>100	
F1						**100**			
F7						**100**			
F12						**100**			
	**bones isolates**								
D14	>100					**100**		>100	
D15(MRSA)						**100**			
D17(MRSA)						**100**			
D20						**100**			
